# Case Report: Severe kidney involvement in a case of very early onset inflammatory bowel disease

**DOI:** 10.3389/fimmu.2025.1684476

**Published:** 2025-11-19

**Authors:** Enrico Drago, Roberta Carfora, Barbara Cafferata, Gabriele Gaggero, Laura Puzone, Edoardo La Porta, Sara Signa, Paolo Gandullia, Valerio Gaetano Vellone, Andrea Angeletti, Serena Arrigo

**Affiliations:** 1 Department of Neuroscience, Rehabilitation, Ophthalmology, Genetics, Maternal and Child Health (DINOGMI), University of Genoa, Genova, Italy; 2 UOC Reumatologia e Malattie Autoinfiammatorie, IRCCS Istituto Giannina Gaslini, Genova, Italy; 3 Pathology Unit, IRCCS Istituto Giannina Gaslini, Genoa, Italy; 4 Division of Nephrology, Dialysis and Transplantation, IRCCS Istituto Giannina Gaslini, Genoa, Italy; 5 Pediatric Gastroenterology and Endoscopy Unit, IRCCS Istituto Giannina Gaslini, Genova, Italy

**Keywords:** VEO-IBD, vedolizumab, ulcerative colitis, tubulointerstitial nephritis (TIN), drug-induced acute interstitial nephritis

## Abstract

**Background:**

Patients affected by very early onset inflammatory bowel disease (VEO-IBD) are frequently refractory to standard treatments. Despite the lack of randomized clinical trials, vedolizumab emerged as an effective and safe alternative treatment in VEO-IBD resistant to TNF antagonists. Here, we present a case of VEO-IBD with ulcerative colitis (UC) phenotype developing renal injury after vedolizumab administration.

**Case presentation:**

An 11-year-old female patient with VEO-UC was referred to our clinic for fever, nausea, and fatigue. She was treated with vedolizumab for 1 year due to steroid-dependent disease and failure of multiple therapies, including anti-TNF agents. At admission, she was in steroid-free clinical and endoscopic remission, with leukocytosis, increased inflammatory markers, and a rise in serum creatinine. Urine samples revealed persistent leukocyturia over the past 8–10 months with the absence of lower urinary tract symptoms and negative serial urine culture. MRI showed swollen-looking kidneys with bilateral irregular Diffusion-Weighted Imaging (DWI) signal restriction. Kidney biopsy revealed the presence of acute tubular damage with a mixed interstitial inflammatory infiltrate consistent with drug-induced acute tubulointerstitial nephritis (TIN). After prompt start of systemic glucocorticoid therapy and temporary discontinuation of vedolizumab, normalization of renal function and urinalysis was observed. Vedolizumab was restarted after 2 months, pre-medicated with steroids. The follow-up renal biopsy performed after 6 months showed a regression of the histological pattern, with chronic signs characterized by mild tubular atrophy and interstitial fibrosis.

**Conclusion:**

Vedolizumab-related acute TIN is a potentially severe complication, rarely described in adult patients. We report the first case of VEO-IBD with a probable vedolizumab-related acute TIN treated with corticosteroids, with a good response and maintenance of vedolizumab. Persisting sterile leukocyturia could represent an early sign.

## Introduction

Inflammatory bowel diseases (IBDs) are chronic inflammatory disorders that affect the digestive tract, including Crohn’s disease (CD), ulcerative colitis (UC), and inflammatory bowel disease-unclassified (IBD-U). Albeit it is traditionally considered a disease of adulthood, there was an increased rate of very early onset IBD (VEO-IBD), defined by the onset of symptoms before the age of 6 years (1). VEO-IBD is sustained by monogenic pathological variants in 8%–32% of cases (2, 3), characterized by inborn errors of immunity (IEI) in more than 70% of genetic cases (4).

Therapeutic failure in VEO-IBD is more frequent than in older pediatric subjects with IBD. In approximately 70% of cases, VEO-IBD is poorly responsive to the standard treatment of IBD, including 5-Aminosalicylic Acid, steroids, and immunomodulators (mercaptopurine, azathioprine, and methotrexate), therefore needing an alternative approach (5). Anti-tumor necrosis factor (anti-TNF) agents have recently shown relative efficacy in patients with VEO-IBD, especially in patients with CD phenotype, although they are not effective in maintaining long-term remission in more than 20% of treated patients (6). Vedolizumab, a gut-selective anti-α4β7 integrin humanized monoclonal antibody, has shown promising results in the treatment of pediatric IBD with primary failure or loss of response to other treatments, especially in UC (7–11). Although it represents a promising rescue therapy, data on safety in the pediatric population should be expanded (10–12).

Here, we present a pediatric case of renal injury due to drug-induced acute tubulointerstitial nephritis (TIN) in an 11-year-old female patient affected by VEO-IBD.

## Case presentation

We present a case involving an 11-year-old female patient diagnosed with VEO-IBD exhibiting UC symptoms, which first manifested at the age of 4. Next-generation sequencing (NGS) genetic panel for genes associated with VEO-IBD was negative (**Supplementary Table 1**). Due to the limited response to first-line therapy (5-ASA, topical steroid, and azathioprine) and steroid-dependent disease, infliximab (IFX) was started at 8 years old without achieving either clinical improvement or mucosal healing despite the maximized dose (8.3 mg/kg every 4 weeks). For this reason, an approach with thalidomide was attempted with subsequent discontinuation after less than 6 months due to the appearance of a thalidomide-related pseudotumor cerebri. When the patient was 9, due to persistent steroid dependence, adalimumab, a different anti-TNF agent, was administered with endoscopic and histological evidence of active disease Ulcerative Colitis Endoscopic Index of Severity after 3 months of therapy. After failure of multiple lines of therapy, vedolizumab was begun with a good clinical and endoscopic response at the optimized dosage of 10 mg/kg every 8 weeks in order to keep levels between 10 and 15 μg/mL during maintenance. Mesalamine was maintained from the diagnosis and associated with every major treatment change.

She was referred to the emergency department for a persisting fever poorly responsive to oral antibiotic therapy, vomiting, fatigue, and loss of weight. Symptoms persisted for 2 weeks before and started a few days after the last infusion of vedolizumab. At admission, laboratory tests showed leukocytosis (White Blood Cells, 16,540 cells/mmc), thrombocytosis (Platelets, 569,000 cells/mmc), increased C-reactive protein (CRP; 10.74 mg/dL), and acute kidney injury (AKI), as well as risk stage according to the RIFLE criteria (serum creatinine increased by 0.4 to 0.7 mg/dL, eGFR 9 mL/min/1.73 m^2^ with revised Schwartz equation). Urine samples revealed leukocyturia (519 cells/μL) with a negative urine culture and without symptoms consistent with lower urinary tract infection. Seriate blood and stool cultures, chest X-ray, and abdominal ultrasound were negative.

She was admitted to the pediatric gastroenterology department for further investigations. Regarding the VEO-IBD, she was in steroid-free clinical remission (Pediatric Ulcerative Colitis Activity Index 0). Pancolonoscopy confirmed the mucosal healing. CT abdomen revealed kidneys with slightly bumpy margins, characterized by a non-homogeneous contrast enhancement with a minimal amount of pelvic fluid. Supposing a culture-negative urosepsis, a urinary tract infection, we administered the antibiotic piperacillin-tazobactam, with no effect in terms of clinical symptoms and serum inflammatory markers, which remained elevated.

**Figure 1:**
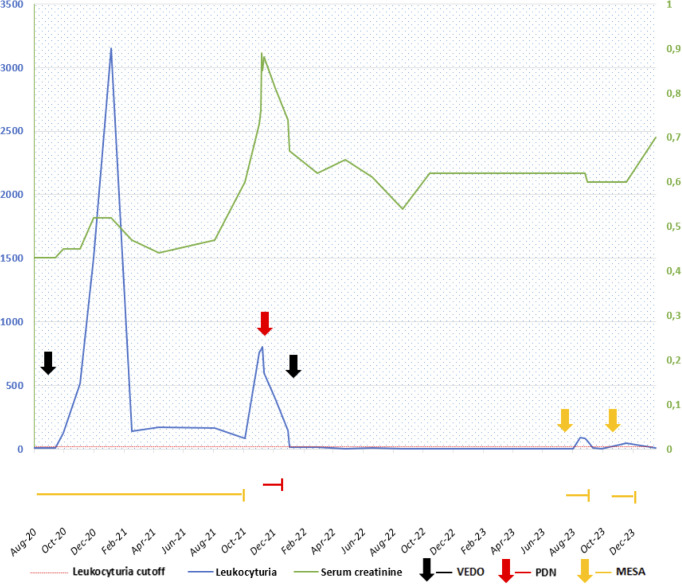
Trends in serum creatinine and leukocyturia over the past 2 years. Black arrows and lines indicate I vedolizumab (VEDO) infusion and restart. Red arrows and lines indicate the start of prednisone (PDN) following tubulointerstitial nephritis (TIN) diagnosis. Orange arrows and lines indicate the treatment with mesalamine (MESA). The dashed horizontal red line indicates the cut-off of normal leukocyturia (<20 cells/μL)..

Reviewing urinalysis and laboratory data, we noted an increase in urinary leukocytes and a really limited rise in serum creatinine also after the first vedolizumab infusion (**Figure 1**). Therefore, due to persistent fever and worsening of renal function (creatinine 0.89 mg/dL, eGFR 67 mL/min/1.73 m^2^), vedolizumab and mesalamine were discontinued due to suspicion of drug-induced nephropathy, and an ultrasound-guided kidney biopsy was performed.

**Figure 2:**
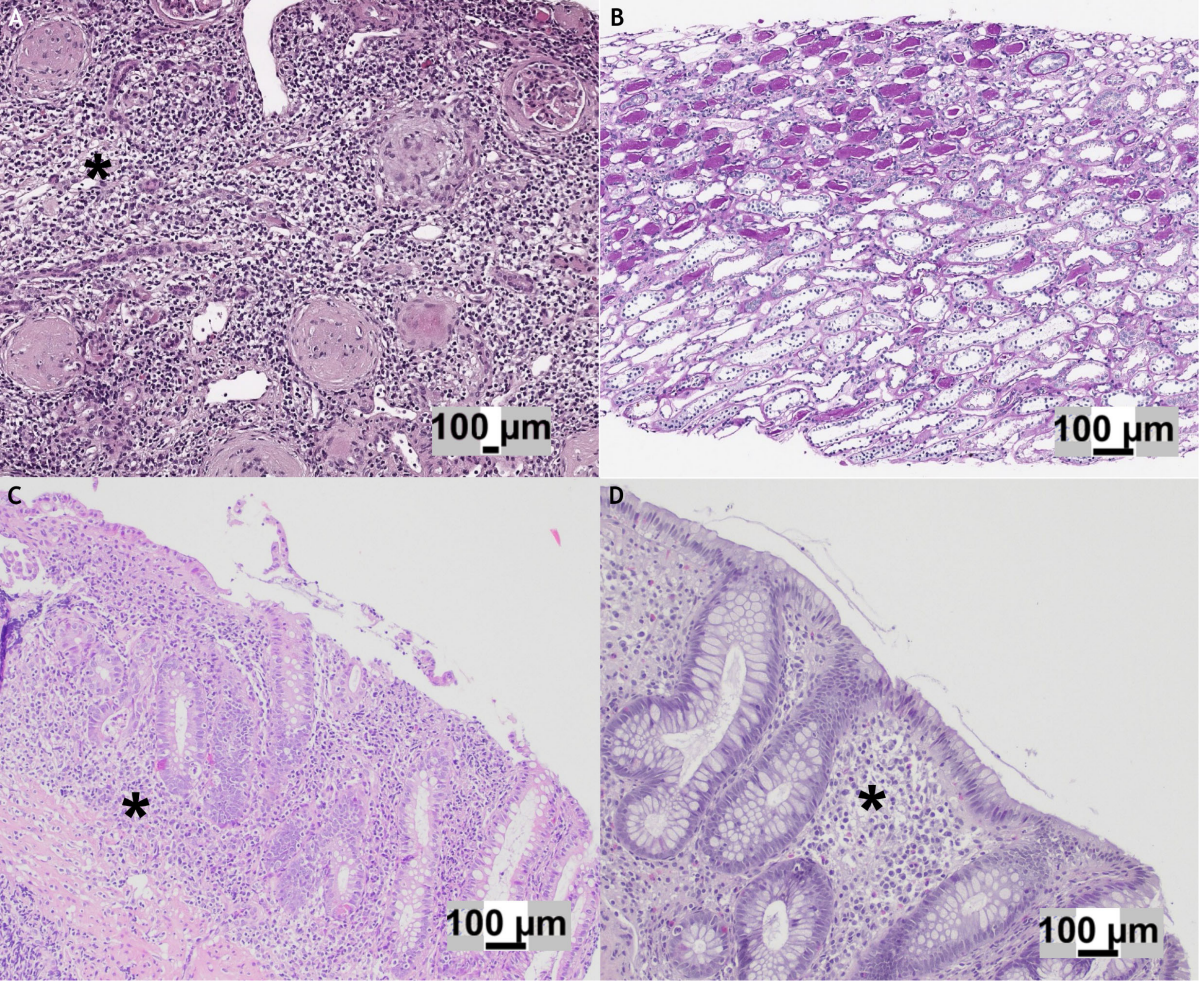
**(A)** Hematoxylin and eosin (H&E) stain of the initial kidney biopsy showing global glomerulosclerosis associated with an interstitial inflammatory infiltrate (*) predominantly composed of lymphocytes. **(B)** H&E stain of the follow-up kidney biopsy demonstrating mild tubular atrophy in the medullary compartment, with no evidence of active inflammation. **(C)** Gastrointestinal biopsy displaying an ulcerative colitis-like pattern, characterized by continuous, marked inflammatory infiltrate (*) with cryptitis and ulcerations. **(D)** Colon biopsy obtained at the time of kidney injury: in comparison with panel C, no active inflammation, cryptitis, or ulcerations were observed, although crypt architectural distortion was present..

Hematoxylin and eosin stain showed diffuse interstitial inflammatory infiltrate and focal tubular atrophy localized mostly in the medullary compartment (**Figure 2A**). The inflammatory infiltrate was composed mostly of T (CD3+) and B (CD20+) lymphocytes, with intermixed histiocytes (CD68+), neutrophils, and rare eosinophils (**Figure 2A**).

More than half of the glomeruli (10/16) were characterized by a fibrous reaction affecting Bowman’s capsule and capillary collapse, and three of them were globally sclerotic. Immunofluorescence was negative (IgA, IgG, IgM, C3, C4, and C1q). The final diagnosis was consistent with acute TIN.

Based on histological findings, anti-inflammatory treatment with prednisone (1 mg/kg) was administered for 1 month and a withdrawal of 2 weeks, with a prompt decrease of serum creatinine and normalization of leukocyturia (**Figure 1**). After 2 months, due to the lack of an effective therapeutic alternative for IBD, vedolizumab therapy was administered combined with glucocorticoids (prednisone 0.5 mg/kg/die from the day before to the day after the infusion). At 1 year of follow-up, the patient received a total of nine vedolizumab infusions combined with the same steroid scheme. Blood and urine tests showed no further worsening of renal function. Following the reintroduction of low-dose mesalamine (35 mg/kg), mild leukocyturia reappeared on two occasions (**Figure 1**). A new kidney biopsy performed at 6 months of follow-up after the previous one demonstrated a regression of the interstitial inflammatory infiltrate and the absence of obsolescent glomeruli (**Figure 2B**).

## Discussion

To the best of our knowledge, we report here the first pediatric case of AKI likely related to vedolizumab-induced TIN in a child with VEO-UC. Following the failure of multiple previous therapies, including two anti-TNF agents, vedolizumab was started with a good clinical response and mucosal healing. After 2 years, she presented with AKI due to drug-related TIN, clinically and histologically responsive to glucocorticoid treatment. The previously applied “long premedication” scheme with the steroid (11) allowed vedolizumab infusions to be restarted without relapses.

VEO-IBD treatment remains highly challenging for the pediatric gastroenterologist, and it needs to be tailored, given the clinical and genetic heterogeneity of this condition. This leads to the requirement for multidisciplinary management and prompt genetic counselling. Although vedolizumab has carved out a role in the treatment of VEO-UC in recent years, data on possible adverse effects in this challenging cohort are certainly needed.

According to the latest evidence (12–14) in pediatric subjects, adverse events mostly correspond to infusion-related reactions (fever, arthralgia/myalgia, headache, and nausea/vomiting) and viral upper airway infections, ranging from 4% to 9%. Severe adverse events leading to drug discontinuation are rare: anaphylaxis, septic arthritis, deep vein thrombosis, and leukocytoclastic vasculitis (14).

Kidney involvement due to vedolizumab is rare, and to the best of our knowledge, AKI related to vedolizumab infusion was not previously described in childhood. A recent systematic review by Forss et al. reported nine adult cases of TIN in patients with IBD receiving vedolizumab (**Table 1**) (15). Clinical presentation was heterogeneous: fever and systemic symptoms occurred in 4/9 cases, while 3/9 were asymptomatic (15). Concomitant aminosalicylates were used in 3/9 (15). Corticosteroids were administered in 8/9, with complete recovery within 4 weeks in 2/9 and persistent renal impairment in 5/9 (**Table 1**). Taken together with our case, these findings underscore the need for baseline and periodic renal function testing (serum creatinine and urinalysis) before and throughout vedolizumab therapy. Kidney biopsies typically showed T lymphocyte-predominant TIN with variable eosinophils and early tubular injury/fibrosis. The tubulointerstitial cellular infiltrate was reported in all cases and was mainly characterized by CD4+ T cells (11, 15–22). As with other drug-induced TIN, the monoclonal antibody may act as a hapten or planted antigen in the tubulointerstitium, triggering a T cell-mediated inflammatory cascade that injures renal tubules (23). Notably, the α4β7 integrin target of vedolizumab has no well-defined role in kidney tissue, suggesting the nephritis arises from off-target immune dysregulation rather than direct integrin blockade (24, 25). Blocking gut-homing α4β7 may alter lymphocyte trafficking or immune homeostasis, potentially triggering misdirected T-cell responses against renal tissue.

**Table 1:** Main clinical reports describing vedolizumab-induced TIN in adults (adapted from Forss et al. (15)).

References	Age/gender	IBD classification	Time from the start of VEDO	TIN treatment	eGFR after TIN (stage)	VEDO restart
*Bailly* et al. (11)	55 F	CD	0 wk	Steroid	63 mL/min/1.73 m^2^ (CKD 2)	Yes
*Subhaharan* et al. (16)	20 M	UC	≈40 wk	Steroid	Normal	No
*Zhang* et al. (17)	33 F	CD	≈8 wk	Steroid	38 mL/min/1.73 m^2^ (CKD 3b)	No
*Simpson* et al. (18)	44 F	CD	≈48 wk	Steroid	44 mL/min/1.73 m^2^ (CKD 3b)	No
*Jahan* et al. (19)	19 F	UC	≈8 wk	Steroid	NA	No
*Kepley* et al. (20)	33 M	UC	NA	Steroid	NA	Yes (failed)
*Kepley* et al.	45 F	UC	NA	NA	NA	NA
*Muzib* et al. (21)	58 F	UC	≈48 wk	Steroid	58 mL/min/1.73 m^2^ (CKD 3a)	NA
*O’Leary* et al. (22)	21 M	UC	>24 wk	Steroid	86 mL/min/1.73 m^2^ (CKD 2)	Yes (failed)

CD, Crohn's disease; CKD, chronic kidney disease; eGFR, estimated Pediatric Ulcerative Colitis Activity Index by 2021 CKD-EPI; IBD, inflammatory bowel disease; UC, ulcerative colitis, wk, weeks; TIN, tubulointerstitial nephritis; VEDO, vedolizumab.

Acute interstitial nephritis is an important cause of kidney injury, causing 3%–7% of AKI in children (26). Urinalysis monitoring allows the early detection of chronic sterile leukocyturia, a known sign of drug-induced TIN (27). Tubulointerstitial involvement in IBD is frequently related to chronic exposure to aminosalicylate or, rarely, may be a possible extraintestinal manifestation of active bowel disease (28, 29). Considering the absence of recent exacerbation of the colitis (**Figures 2C, D**) and the lack of granuloma evidence on renal biopsy (**Figures 2A, B**), we reasonably exclude that the TIN manifested itself as an extraintestinal manifestation of IBD.

As a limitation of our case, we report the chronic exposure of the patient to mesalamine, which was discontinued at the AKI. The reintroduction of mesalamine therapy was correlated with a slight rise in leukocyturia, with subsequent discontinuation, even considering the clinical remission. Nevertheless, the extended duration of 5-ASA treatment without any adverse effects and the significant temporal association between the start of vedolizumab and the onset of leukocyturia (**Figure 1**) led us to lean toward attributing the tubulointerstitial damage to the biological therapy rather than the exposure to aminosalicylates (29), or at least to a cumulative effect of the two drugs.

In conclusion, we report the first case of VEO-IBD with a probable vedolizumab-related acute TIN. Despite severe kidney damage, steroid therapy was effective, with functional recovery, normalization of the urinalysis, and resumption of vedolizumab infusions without relapses. Persistence of sterile leukocyturia may represent an early sign of drug-related TIN.

## Data Availability

The original contributions presented in the study are included in the article/**Supplementary Material**. Further inquiries can be directed to the corresponding author.

## References

[1] Benchimol EI Fortinsky KJ Gozdyra P Van den Heuvel M Van Limbergen J Griffiths AM . Epidemiology of pediatric inflammatory bowel disease: a systematic review of international trends. Inflammation Bowel Dis. (2011) 17:423–39. doi: 10.1002/ibd.21349, PMID: 20564651

[2] Charbit-Henrion F Parlato M Hanein S Duclaux-Loras R Nowak J Begue B . Diagnostic Yield of Next-generation Sequencing in Very Early-onset Inflammatory Bowel Diseases: A Multicentre Study [published correction appears in J Crohns Colitis. 2021 Mar 5;15(3):517-518. J Crohns Colitis. (2018) 12:1104–12. doi: 10.1093/ecco-jcc/jjy068, PMID: 29788237 PMC6113703

[3] Collen LV Kim DY Field M Okoroafor I Saccocia G Whitcomb SD . Clinical phenotypes and outcomes in monogenic versus non-monogenic very early onset inflammatory bowel disease. J Crohns Colitis. (2022) 16:1380–96. doi: 10.1093/ecco-jcc/jjac045, PMID: 35366317 PMC9455789

[4] Ouahed J Spencer E Kotlarz D Shouval DS Kowalik M Peng K . Very early onset inflammatory bowel disease: A clinical approach with a focus on the role of genetics and underlying immune deficiencies. Inflammation Bowel Dis. (2020) 26:820–42. doi: 10.1093/ibd/izz259, PMID: 31833544 PMC7216773

[5] Kelsen JR Conrad MA Dawany N Patel T Shraim R Merz A . The unique disease course of children with very early onset-inflammatory bowel disease. Inflammation Bowel Dis. (2020) 26:909–18. doi: 10.1093/ibd/izz214, PMID: 31560377 PMC7216772

[6] Kerur B Fiedler K Stahl M Hyams J Stephens M Lu Y . Utilization of antitumor necrosis factor biologics in very early onset inflammatory bowel disease: A multicenter retrospective cohort study from North America. J Pediatr Gastroenterol Nutr. (2022) 75:64–9. doi: 10.1097/MPG.0000000000003464, PMID: 35622080 PMC12034324

[7] Tyler CJ Guzman M Lundborg LR Yeasmin S Zgajnar N Jedlicka P . Antibody secreting cells are critically dependent on integrin α4β7/MAdCAM-1 for intestinal recruitment and control of the microbiota during chronic colitis. Mucosal Immunol. (2022) 15:109–19. doi: 10.1038/s41385-021-00445-z, PMID: 34433904 PMC8732264

[8] Rogler G . Mechanism of action of vedolizumab: do we really understand it? Gut. (2019) 68:4–5. doi: 10.1136/gutjnl-2018-316777, PMID: 30077996

[9] Shah P McDonald D . Vedolizumab: an emerging treatment option for pediatric inflammatory bowel disease. J Pediatr Pharmacol Ther. (2021) 26:795–801. doi: 10.5863/1551-6776-26.8.795, PMID: 34790068 PMC8592007

[10] Garcia-Romero R Martinez de Zabarte Fernandez JM Pujol-Muncunill G Donat-Aliaga E Segarra-Cantón O Irastorza-Terradillos I . Safety and effectiveness of vedolizumab in paediatric patients with inflammatory bowel disease: an observational multicentre Spanish study [published correction appears in Eur J Pediatr. 2021 May 23. Eur J Pediatr. (2021) 180:3029–38. doi: 10.1007/s00431-021-04063-6, PMID: 33880650

[11] Bailly E Von Tokarski F Beau-Salinas F Picon L Miquelestorena-Standley E Rousseau G . Interstitial nephritis secondary to vedolizumab treatment in crohn disease and safe rechallenge using steroids: A case report. Am J Kidney Dis. (2018) 71:142–5. doi: 10.1053/j.ajkd.2017.08.008, PMID: 29162338

[12] Atia O Shavit-Brunschwig Z Mould DR Stein R Matar M Aloi M . Outcomes, dosing, and predictors of vedolizumab treatment in children with inflammatory bowel disease (VEDOKIDS): a prospective, multicentre cohort study. Lancet Gastroenterol Hepatol. (2023) 8:31–42. doi: 10.1016/S2468-1253(22)00307-7, PMID: 36306803

[13] Fabiszewska S Derda E Szymanska E Osiecki M Kierkus J . Safety and effectiveness of vedolizumab for the treatment of pediatric patients with very early onset inflammatory bowel diseases. J Clin Med. (2021) 10:2997. doi: 10.3390/jcm10132997, PMID: 34279480 PMC8268556

[14] Fang S Song Y Zhang C Wang L . Efficacy and safety of vedolizumab for pediatrics with inflammatory bowel disease: a systematic review. BMC Pediatr. (2022) 22:175. doi: 10.1186/s12887-022-03229-x, PMID: 35379216 PMC8978350

[15] Forss A Flis P Sotoodeh A Kapraali M Rosenborg S . Acute interstitial nephritis in patients with inflammatory bowel disease treated with vedolizumab: a systematic review. Scand J Gastroenterol. (2024) 59:821–9. doi: 10.1080/00365521.2024.2345383, PMID: 38682791

[16] Subhaharan D Ramaswamy PK Francisco S Ishaq N . Vedolizumab-induced acute interstitial nephritis in ulcerative colitis. ACG Case Rep J. (2022) 9:e00788. doi: 10.14309/crj.0000000000000788, PMID: 35765681 PMC9232362

[17] Zhang PL Pancioli T Li W Kanaan HD . Electron microscopic findings can support multiple etiologies of nephrotoxicity in renal tubules. Ultrastruct Pathol. (2020) 44:481–8. doi: 10.1080/01913123.2020.1839152, PMID: 33131373

[18] Simpson N Seenan JP Patel R Kipgen D . Acute interstitial nephritis secondary to vedolizumab. BMJ Case Rep. (2021) 14:e243568. doi: 10.1136/bcr-2021-243568, PMID: 34799387 PMC8606757

[19] Jahan S Xu A Danner R Sellars I Coates TP . Acute interstitial nephritis with inflammatory bowel disease. Nephrology. (2022) 27:69–70.

[20] Kepley A Marino D DeCross A . P053 Interstitial nephritis from IBD: complicated conclusions. Am J Gastroenterol. (2021) 116:S14. doi: 10.14309/01.ajg.0000798812.13521.04, PMID: 37461971

[21] Muzib A Parikh R Bijol V Uppal NN Sachdeva M Vedolizumab-induced acute interstitial nephritis and acute tubular necrosis: PO2260. J Am Soc Nephrol. (2020) 31:688. doi: 10.1681/ASN.20203110S1688c

[22] O’Leary C Wong D Wilson G . Vedolizumab-induced acute interstitial nephritis with failure of steroid prophylaxis on vedolizumab rechallenge. BMJ Case Rep. (2023) 16:e254715. doi: 10.1136/bcr-2023-254715, PMID: 37699736 PMC10503377

[23] Krishnan N Perazella MA . Drug-induced acute interstitial nephritis: pathology, pathogenesis, and treatment. Iran J Kidney Dis. (2015) 9:3–13., PMID: 25599729

[24] McLean LP Cross RK . Pharmacodynamic assessment of vedolizumab for the treatment of ulcerative colitis. Expert Opin Drug Metab Toxicol. (2016) 12:833–42. doi: 10.1080/17425255.2016.1181171, PMID: 27096357 PMC4917449

[25] Pozzi A Zent R . Integrins in kidney disease. J Am Soc Nephrol. (2013) 24:1034–9. doi: 10.1681/ASN.2013010012, PMID: 23641054 PMC3699832

[26] Greising J Trachtman H Gauthier B Valderrama E . Acute interstitial nephritis in adolescents and young adults. Child Nephrol Urol. (1990) 10:189–95., PMID: 2088589

[27] Perazella MA Markowitz GS . Drug-induced acute interstitial nephritis. Nat Rev Nephrol. (2010) 6:461–70. doi: 10.1038/nrneph.2010.71, PMID: 20517290

[28] Angeletti A Arrigo S Madeo A Molteni M Vietti E Arcuri L . Different renal manifestations associated with very early onset pediatric inflammatory bowel disease: case report and review of literature. BMC Nephrol. (2021) 22:146. doi: 10.1186/s12882-021-02358-2, PMID: 33888087 PMC8061217

[29] Moss JG Parry CM Holt RCL McWilliam SJ . 5-ASA induced interstitial nephritis in patients with inflammatory bowel disease: a systematic review. Eur J Med Res. (2022) 27:61. doi: 10.1186/s40001-022-00687-y, PMID: 35488310 PMC9052675

