# Supplementary material for: Case Report: Severe kidney involvement in a case of very early onset inflammatory bowel disease
Source: Front Immunol. 2025 Nov 19;16:1684476. doi: 10.3389/fimmu.2025.1684476 (PMC12672911; doi:10.3389/fimmu.2025.1684476)
Supplement: Supplementary file 1 [file Table1.docx]

**NGS custom panel for VEO-IBD (135 genes):**
ABCB1, ADA, ADAM17, AICDA, AIRE, ALPI, ANGPTL3, ANKZF1, APOB, ARPC1B, BACH2, BTK, CARD8, CARMIL2, CASP8, CD3D, CD3E, CD3G, CD40LG, CD55, CLMP, COG6, COL7A1, CTLA4, CYBA, CYBB, DCLRE1C, DEF6, DGAT1, DKC1, DOCK8, DUOX2, EPCAM, FERMT1, FLNA, FOXP3, G6PC3, GUCY2C, HPS1, HPS4, HPS6, HSPA1L, ICOS, IKBKG, IL10, IL10RA, IL10RB, IL12RB1, IL21, IL2RA, IL2RB, IL2RG, IL6, IL7R, INAVA, IRGM, ITCH, ITGB2, JAK1, JAK3, LACC1, LCT, LIG4, LRBA, MALT1, MASP2, MEFV, MTTP, MVK, MYO5B, NCF2, NCF4, NEUROG3, NFAT5, NFKB1, NFKBIA, NHEJ1, NLRC4, NLRP12, NOD2, NOX1, NPC1L1, PCSK1, PCSK9, PIK3CD, PIK3R1, PLCG2, PNLIP, PNP, PTPRC, RAG1, RAG2, RBCK1, RELA, RIPK1, RNF31, RTEL1, SAR1B, SBDS, SH2D1A, SI, SKIV2L, SLC10A2, SLC26A3, SLC37A4, SLC39A4, SLC5A1, SLC9A3, SLCO2A1, SPINT2, STAT1, STAT3, STAT5B, STIM1, STX3, STXBP2, TCN2, TERT, TGFB1, TGFBR1, TGFBR2, TINF2, TMPRSS15, TNFAIP3, TNFRSF13B, TRIM22, TRNT1, TTC37, TTC7A, UBR1, WAS, WRAP53, XIAP, ZAP70, ZBTB24.
